# The Bioactivity and Phytochemicals of *Pachyrhizus erosus* (L.) Urb.: A Multifunctional Underutilized Crop Plant

**DOI:** 10.3390/antiox11010058

**Published:** 2021-12-27

**Authors:** Varun Jaiswal, Shweta Chauhan, Hae-Jeung Lee

**Affiliations:** 1 Department of Food and Nutrition, College of BioNano Technology, Gachon University, Seongnam-si 13120, Korea; computationalvarun@gmail.com (V.J.); chauhanshweta210@yahoo.com (S.C.); 2 Institute for Aging and Clinical Nutrition Research, Gachon University, Seongnam-si 13120, Korea; 3 Department of Health Sciences and Technology, GAIHST, Gachon University, Incheon 21999, Korea

**Keywords:** antioxidants, flavonoids, *Pachyrhizus erosus*, jicama, phytochemicals, pharmacological activities, anticancer, anti-diabetes

## Abstract

*Pachyrhizus erosus* (L.) Urb. is an underutilized crop plant belonging to the Fabaceae family. In recent years, the plant received huge attention and was introduced in different countries owing to properties such as a high nutritional content, its nitrogen-fixing abilities, and different biological activities such as its antioxidant, immune modulation, anticancer, anti-diabetes, anti-osteoporosis, antiviral, and antiaging affects, among others. In this review, an attempt has been made to comprehensively compile the biological activities of the plant to provide a panoramic view of the current efforts and further directions, which may lead to the development of pharmacological applications. This information will be helpful in creating interest towards *P. erosus* and it may be useful in developing the plant for medical applications and/or as a functional food. More than 50 phytochemicals have been reported from the plant, which belong to different chemical classes such as triterpenoids, organic acid, flavonoids, and fatty acids. Numerous biological activities were reported from the plant through in vivo, in vitro, ex vivo, and human studies. However, well-defined clinical studies are still lacking for the establishment of any biological properties that could be further developed. Suggestions for the further development of *P. erosus*, according to current knowledge about the different biological properties, has also been provided.

## 1. Introduction

The positive roles of antioxidant and immune modulation properties against infectious, as well as non-infectious, diseases have emphasized the importance of dietary plants with these properties [1,2,3]. *Pachyrhizus erosus* (*P. erosus*) is primarily a crop plant, which is underutilized in different parts of the world despite having a high nutritional content. Like the traditional medicinal plants, it is used in folk medicine and is known for its antioxidant properties [4,5,6,7,8]. Different parts of the plant have pharmacological and health-promoting properties, which have the potential to be utilized in different biological activities and diseases, such as antioxidants, immune modulation, anti-aging effects, diabetes, cancer, osteoporosis, and viral and fungal diseases [6,9,10,11,12,13,14,15,16,17].

*P. erosus* is also known as jicama, yam bean, Mexican turnip, shankhalu (in Bengali), singkamas (in the Philippines), kuzu-imo (in Japan) and bang kuang (in Korea and China).

*P. erosus* originated from the semiarid tropics of Central America and Mexico, and from there the cultivation of *P. erosus* expanded into different parts of the world, such as the Philippines, Indonesia, Malaysia, Vietnam, Laos, Thailand, Taiwan, Cambodia, Singapore, Burma, China, and India. In the recent past, *P. erosus* was introduced into South Korea [18,19,20]. Considering its high nutrient content and nitrogen-fixing properties, *P. erosus* was also introduced into the African countries with the objective to complement food sources and improve the sustainability of the farming structure so that it might overcome food insecurity. It can also counter malnutrition found in children under five years [21] in some African countries, and is mainly endorsed due to the extra dependency on the popular tuber and root crops, such as sweet potato, potato, and cassava, which have limited sources of absorbable micronutrients and proteins.

*P. erosus* is still an underutilized crop, but due to its high nutrient content, its nitrogen-fixing properties for sustainable farming, its different pharmacological properties, and the successful introduction of *P. erosus* in different countries around the world, the production of *P. erosus* is expected to grow in the near future. It can produce heavy storage roots compared to other comparable root crops, such as cassava (*Manihot esculenta*), as well as having several times more protein content [22].

The seeds of *P. erosus* are toxic due to the presence of rotenone. Rotenone is considered as a moderately toxic compound for humans by the World Health Organization. The ingestion of seeds can cause severe toxicity, which may lead to death [23,24,25,26]. Therefore, the seeds cannot be consumed directly. However, the use of *P. erosus* in folk medicine has been reported [8,27]. Therefore, researchers have explored the presence of dietary fiber, protein, carbohydrates, inulin, vitamin C, folates, riboflavins, pyridoxine, pantothenic acid, thiamin, and other phytochemicals in the tubers of *P. erosus*, which has provided the basis of its pharmacological potential [6,9,10,11,12,13,14,15,16,17]. Similarly, the seeds and leaves are also rich in different phytochemicals, which further encourages researchers to develop natural drugs and cosmetics [4,9,10,13]. Numerous studies have reported the potential application of *P. erosus* in a number of diseases, as well as immune enhancement, which not only suggests the potential of the plant to be developed as a medicinal plant but also shows it as an underutilized crop. Although the increased production of *P. erosus* is expected in the near future, a comprehensive compilation of its pharmacological properties and phytoconstituents is missing for the plant in the literature. The current review is an attempt to assemble the information of phytochemicals and biological activities of different parts of this perennial plant, which may pave the way for the development of a natural pharmacological intervention for different diseases, as well as promoting health and improving cosmetics.

## 2. Electronic Literature Search

An extensive literature search was carried out through online databases, such as PubMed, Scopus, Google Scholar, Google, and ReseachGate. It included important keywords and their combinations according to previous studies, such as *Pachyrhizus erosus*, jicama, yam bean, Mexican turnip, kuzu-imo, bang kuang, nutrient composition, phytochemical composition, pachyrhizus and biological activity, *Pachyrhizus erosus* and cancer, *P. erosus* and review, etc., which were used for the literature search. The resulting research articles, review articles, books, and theses until 2021 were explored and the important literature was included in the current study.

## 3. Botanical Description

*P. erosus* is a perennial vigorous climbing herb that belongs to the Fabaceae family. The plant grows in a warm, humid, and tropical climate from sea level to 1400 m above sea level, with an optimal temperature range of 20 to 28 °C in areas with moderate rainfall, that is, with an average annual rainfall of about 1500 mm. It prefers full sunlight and saturated, moist, well-drained, light, sandy loam, as well as alluvial or volcanic soils. It can produce annual stems 2–6 m long from a tuberous root stock. The root can weigh up to 20 kg. It has been reported that the formation of flowers and tubers occurs almost simultaneously during the development of the plant [19]. It is a herbaceous vine that has compound leaves of a dark green color with a wide variety of leaf shapes, from serrated to serrated fingered. *P. erosus* produces bisexual flowers 1–2.5 cm long which are self-pollinating in nature. Flowering begins 58–68 days after sowing and lasts 92–103 days. It was found that the stigma becomes receptive at 12 h before the opening of the flower, and lasts for 18 h after opening. The appearance is determined by the absence of hairs on the petals, the number of flowers (4–11) on the lateral axis of the inflorescence, i.e., complex racemes, and the length of the inflorescence, which is between 8 and 45 cm. In addition, the morphological features of the legumes (pods), both qualitative and quantitative, are used to separate the species. The size (6–13 cm × 8–17 mm) and reduction of strigous hairs with maturation and color (from pale brown to olive green/brown) are characteristic of *P. erosus* legumes. The seed characters are also specific; they include a color that varies from olive green to brown or reddish-brown, and the shape is flat and square to round, but never reniform [19].

## 4. Nutritional Composition

Primarily, the tuber of *P. erosus* is used for consumption, as the food consists of the underground roots, whereas the seeds of the plant are considered toxic because of the presence of rotenone, which may cause death [23,24,25,26]. Researchers have been also trying to harvest the potential of the seeds to consume as food, due to their high protein and lipid content [28,29].

### 4.1. The Nutritional Composition of the Tuber

The high nutritional components and low anti-nutritional factors show the usefulness of the tuber of *P. erosus* in malnourished regions of the world [30]. The nutritional composition of the tuber may vary according to the form which is consumed, such as its flour, the raw tuber, or its juice. The harvest time can also change the nutritional composition of the tuber [30]. The nutritional composition also depends on the environmental conditions of the surroundings of the plant, and small changes are expected in different studies.

In a study conducted on the fresh tuber, the *P. erosus* tuber had the highest percentage of moisture (82.01 ± 2.24), reducing sugar (1.83 ± 0.22), and crude fiber (1.4 ± 0.14), and the lowest content of starch (9.04 ± 0.11), sucrose (3.24 ± 0.13), carbohydrate (14.9 ± 0.04), total soluble sugar (2.13 ± 0.11), energy (39 ± 1.23 Kcal/100 gm), and ash (0.5 ± 0.12) in comparison with the potato and sweet potato. The lipids (0.1 ± 0.04) and high protein (1.23 ± 0.13) content was also observed in the tuber [30].

Furthermore, vitamins such as ascorbic acid (14 ± 0.1 mg/100 g), thiamine (0.05 ± 0.001 mg/100 g), riboflavin (0.02 ± 0.002 mg/100 g), pyridoxine (0.25 ± 0.01 mg/100 g), niacin (0.2 ± 0.01 mg/100 g) and folic acid (0.001 ± 0.0002 mg/100 g) were also identified in the tuber. The contents of important minerals such as Ca (16 ± 0.45 mg/100 g), Cu (0.048 ± 0.01 mg/100 g), Fe (1.4 ± 0.03 mg/100 g), Mg (12.9 ± 1.05 mg/100 g), Mn (0.06 ± 0.002 mg/100 g), P (18 ± 1.02 mg/100 g), K (172 ± 3.14 mg/100 g), Na (35 ± 1.23 mg/100 g), Zn (0.16 ± 0. 02 mg/100 g), and Se (0.7 ± 0.07 µg/100 g) were also calculated in the tuber of *P. erosus*.

Ten essential and seven non-essential amino acids were detected in the tuber and the content of essential and non-essential amino acids was found to be 12.14 and 28.84 µM/gm, respectively [30].

### 4.2. The Nutritional Composition of the Seeds

*P. erosus* seeds are toxic due to the presence of rotenone; therefore, they cannot be consumed directly. However, high contents of proteins, lipids, Fe, and Ca are observed in the seeds as compared to other legume plants. A study showed that the amino acid (isoleucine, leucine, lysine, methionine, phenylalanine, tyrosine, threonine, valine, and histidine) composition had a better balance of essential amino acids compared to other seeds and concluded that *P. erosus* seeds could be a good source of highly nutritional quality proteins [31]. Different mitigation strategies have been studied for the degradation of rotenone in the seeds, which include drying, roasting, boiling, frying, and alcohol extraction. Drying and roasting were found to be most efficient methods for the degradation, which can degrade up to 80% of rotenone in the seeds [29]. However, the potential for the consumption of the seeds remains very limited, as a rotenone level in the seeds of *P. erosus* did not go into the safe range. In future studies, the combination of different methods may be helpful to reduce the rotenone to safe levels [29].

*P. erosus* seeds, on a wet basis, was analyzed by researchers for their proximate composition, minerals, protein fractions, antinutritional factors, and rotenoids [28]. The seeds showed a high content of protein (28.27 g/100 g), lipids (26.8 g/100 g), ash (4.58 g/100 g), crude carbohydrate (26.85 g/100 g), fiber (6.2 g/100 g) and moisture content (7.30 g/100 g) [28]. The oil content of the seed was reported to be around 20.5% to 28%, which constituted of palmitic (26.7%), stearic (5.7%), oleic (33.4%), linoleic (34.2%), and linolenic (in trace amount) acids [32]. The researchers suggested that the oil could be used in the edible oil industry as a vegetable/cottonseed oil [32]. The quantification of fatty acids in comparison with the other species of the genus *Pachyrhizus* was done in 1999, which showed a total protein content of 29.9% of the seed weight. The fatty acid content was 24% and was found to be made up of palmitic acid (27.8%), stearic acid (4.5%), oleic acid (25.4%), linoleic acid (37.0%), and linolenic acid (1.3%). The total amount of tocopherols was found to be 443.7 mg/kg, which constituted of α-tocopherol (4.8%), γ-tocopherol (94.5%), and δ-tocopherol (0.7%) [33]. The presence of high levels of palmitic acid with low levels of linolenic acid in the seed suggested this as an alternative source of palmitic acid oil in the food industry [33]. A recent study has also shown that the methyl esters of *P. erosus* seed oil can be used as biodiesel in warmer climate countries [34]. While the seeds are rich in protein and fat, they also contain different important minerals. The mineral composition of *P. erosus* is Ca (356.0 mg/100 g), Fe (16.0 mg/100 g), K (992.0 mg/100 g), P (286.0 mg/100 g), Na (6.8 mg/100 g), Cu (1.2 mg/100 g), and Zn (4.0 mg/100 g) [28].

### 4.3. The Nutritional Composition of Seed Flour

The seeds showed a high content of protein (51.20 g/100 g), lipids (1.51 g/100 g), ash (3.64 g/100 g), crude carbohydrate (35.78 g/100 g), fiber (4.42 g/100 g), and moisture content (3.45 g/100 g) [28]. The composition of essential amino acids included isoleucine (4.4 ± 0.16 g/100 g), threonine (3.5 ± 0.07 g/100 g), phenylalanine (5.5 ± 0.38 g/100 g), tyrosine (3.2 ± 0.31 g/100 g), valine (4.6 ± 0.02 g/100 g), lysine (6.5 ± 0.06 g/100 g), histidine (4.1 ± 0.37 g/100 g), methionine (1.4 ± 0.11 g/100 g), and leucine (7.7 ± 0.21 g/100 g). The composition of non-essential amino acids constituted of serine (4.3 ± 0.05 g/100 g), aspartic acid (9.7 ± 0.08 g/100 g), alanine (4.0 ± 0.03 g/100 g), glycine (3.8 ± 0.04 g/100 g), arginine (4.5 ± 0.20 g/100 g), glutamic acid (15.8 ± 0.17 g/100 g), and proline (4.1 ± 0102 g/100 g) [28]. In the seed flour, the toxic compound rotenone was quantified as 0.06 mg/100 g and anti-nutritional compounds, namely tannins, were found to be 6 mg/100 g.

## 5. Phytoconstituents

**Figure 1:**
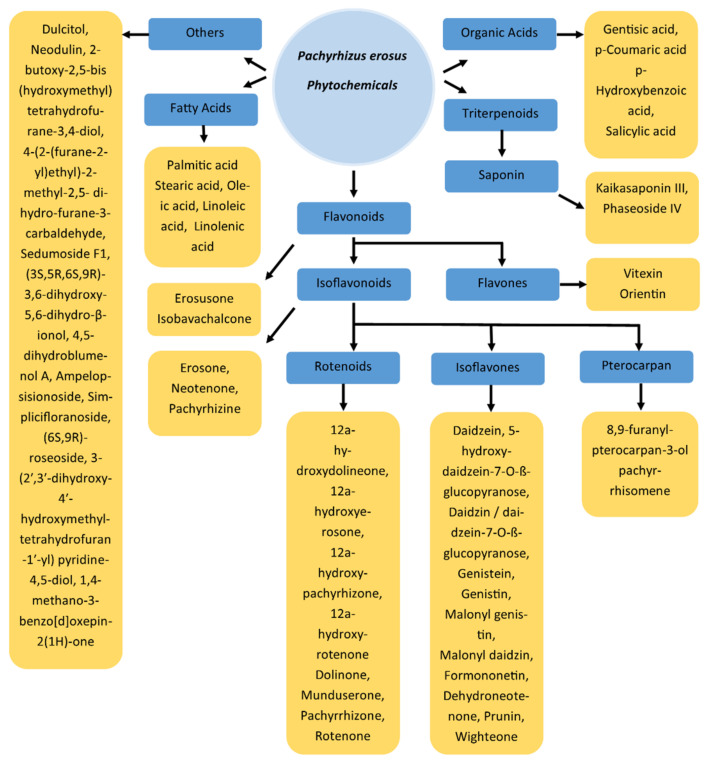
Phytochemicals reported form *P. erosus*.

Researchers have isolated and identified different phytochemicals, mainly from the seeds, tubers, and leaves of *P. erosus* in different studies. In most of the studies, the major motivation behind the phytoconstituent analysis was the possible biological properties of the plant. Therefore, the biological properties of isolated and characterized phytochemicals were also analyzed in most of the studies. Some of the identified phytochemicals were found to be the reason for the biological activity of *P. erosus* as having anticancer, antifungal, and antiviral properties, among others (discussed in the next section). Most of the phytochemicals from *P. erosus* can be grouped into the flavonoids category, although other classes of molecules, such as triterpenes, different organic acids, fatty acids, and volatile organic compounds were also characterized from the different parts of the plant in several studies (Table 1 and Figure 1). These studies are discussed in the subsequent text and Table 1.

In the late 1990s, two triterpenoid glycosides, kaikasaponin III and phaseoside IV, together with daidzin and (+) -abrin, were isolated from the tuber (in both the peel and the flesh) of *P. erosus*. It was the first study in which both triterpienoids, as well as isoflavonoids, were identified in *P. erosus*. The proton (1H) and carbon-13 (13C) nuclear magnetic resonance (NMR) imaging was used for the spectral characterization of the compounds [35].

Around the same time, using bioactivity-guided fractionation on the seeds of *P. erosus*, nine isoflavonoids were isolated, including novel compounds such as coumaronochromene and pachyrrhisomene, as well as known compounds such as pterocarpan, neodulin, 3-arylcoumarin, pachyrrhizin, and six known rotenoids, namely rotenone, munduserone, 12a-hydroxyrotenone, 12a-hydroxydolineone, 12a-hydroxypachyrrhizone and 12a-hydroxyerosone. These compounds were characterized through 1H and 13C NMR imaging [10]. Later, the quantification of pachyrrhine and rotenone in the seeds was validated and the concentration of pachyrhhizin per gram of seeds was found to be in the range of 0.25 mg to 5 mg. Rotenone was observed between 0.58 mg/g and 4 mg/g [36].

In the early 2000s, researchers explored the seeds of *P. erosus* for their phytochemicals to investigate their antifungal properties. In the study, they found nine compounds from the seeds which consisted of five rotenoids (dolineone, pachyrrhizone, 12a-hydroxydolineone, 12a-hydroxypachyrrhizone, and 12a-hydroxyrotenone), two isoflavonoids (neotenone and dehydroneotenone), one phenylfuranocoumarin (pachyrrhizine), and a monosaccharide (dulcitol) [9].

Later in another study, the seed extract was chromatographed for the isolation of phytochemicals, mainly isoflavonoids, present in the seeds. Five compounds were characterized in the dichloromethane extract, which were rotenone, erosone, pachyrrhizone, dolinone, and pachyrrhizine. One compound, i.e., dehydroneotenone, was isolated from the acetone extract of the seeds. The component identity was determined using UV-Vis, IR, 1H and 13C NMR [13].

In the search for antioxidants and skin whitening activities of the tuber of *P. erosus*, researchers identified six active compounds in the ethyl acetate fraction. The identified compounds were daidzein, daidzin, genistin, (8,9)-furanyl-pterocarpan-3-ol, 4-(2-(furane-2-yl)ethyl)-2-methyl-2,5-dihydro-furane-3-carbaldehyde, and 2-butoxy-2,5-bis(hydroxymethyl)-tetrahydrofurane-3,4-diol [37].

In another study by Lukitaningsih and Holzgrabe in 2014, three isoflavonoids (daidzein, daidzein-7-*O*-β-glucopyranose, 5-hydroxy-daidzein-7-*O*-β-glucopyranose), and a new pterocarpan (8,9-furanyl-pterocarpan-3-ol) with antioxidant activities were reported from the tuber of *P. erosus* [6]. Furthermore, in an HPLC-based method, the concentrations of daidzein and genistein were found to be 110.454 and 165.530 mg/100 g, respectively, in the tuber.

Later, researchers again isolated four isoflavonoids, i.e., rotenone, dolineone, 12a-hydroxypachyrhizone, and pachyrizine through column chromatography from the acetone extract of *P. erosus* seeds. The reported yields were 0.01%, 0.006%, 0.004%, and 0.006% for rotenone, dolineone, 12a-hydroxypachyrhizone, and pachyrizine, respectively [38].

In a recent study, the methonolic extract of the leaves of *P.erosus* was used for the isolation and identification of phytochemicals. In this study, two new compounds, erosusone (prenylated chalcone) and 3-episedumoside F_1_ (megastigmane glycoside epimer), along with 13 known compounds were found [39]. The other known compounds were five flavonoids (isobavachalcone, vitexin, wighteone, pruning, and orientin), one 3-benzoxepine lactone (1,4-methano-3-benzo[d]oxepin-2(1*H*)-one), one pyridine-4,5-diol derivative (3-(2′,3′-dihydroxy-4′-hydroxymethyltetrahydrofuran-1′-yl) pyridine-4,5-diol), six megastigmanes, and megastigmane glycosides (sedumoside F_1_, (3*S*,5*R*,6S,9*R*)-3,6-dihydroxy-5,6-dihydro-β-ionol, 4,5-dihydroblumenol A, ampelopsisionoside, simplicifloranoside, and (6*S*,9*R*)-roseoside) [39]. The methods used for structure elucidation were high-resolution electrospray ionization (HR-ESI)-MS, and one-dimensional (1D) and two-dimensional NMR (2D-NMR) spectroscopy [39].

### Volatile Compounds

Researchers have studied the volatile organic compounds present in the leaves of *P. erosus* through gas chromatography coupled with mass spectroscopy and have identified 21 volatile organic compounds present in the leaves (Table S1 Supplementary Materials). *P. erosus* has more volatile compounds compared to other nearby species, i.e., *P. ferrugineus*. *P. erosus* contains mainly aldehydes, ketones, and alcohols among its volatile organic compounds [40].

In a recent study, several important bioactive compounds from the leaves of *P. erosus*, including gentisic acid, p-hydroxybenzoic acid, p-coumaric acid, salicylic acid, L-phenylalanine, and malonyl daidzin were identified. The concentrations of the compounds were found to vary with the applied light source [4] which suggests that the optimal concentration of important phytoconstituents can be achieved through artificial light.

## 6. Bioactivities (In Vitro and In Vivo)

**Figure 2:**
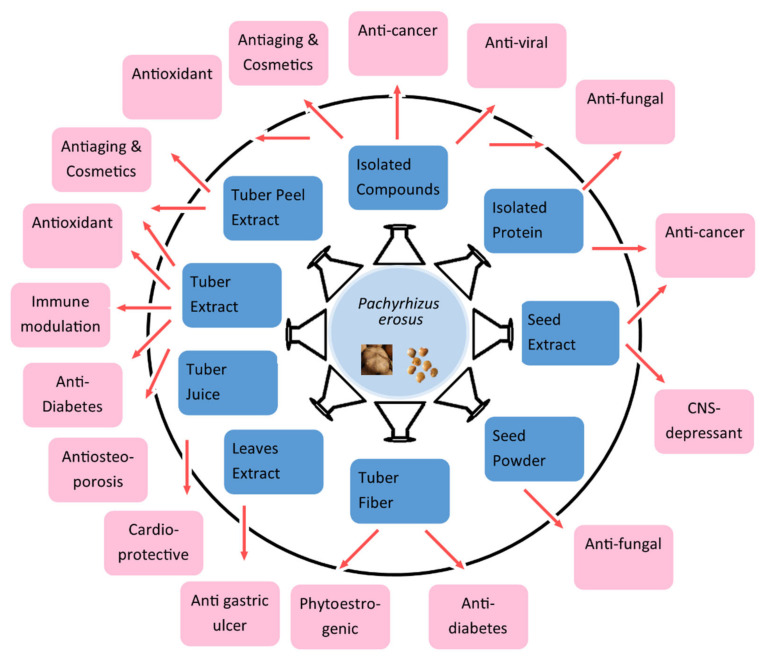
Different biological activities of *P. erosus* through different extracts and forms reported in literature.

Researchers have isolated and identified different phytochemicals, mainly from the seeds, tubers, and leaves. An indication of the pharmacological activities of *P. erosus* was known from ancient times and has been reported in the literature, which has been further explored in research performed mostly in the last two decades. The biological activities against different diseases, and its other pharmacological properties, makes *P. erosus* a probable candidate for further development (Figure 2). This systematic review highlights the different activities and possible directions to pursue further in relation to research on the development of therapeutics. The associated gaps and challenges are discussed in this section. Different parts of the plant have been used for bioactivities in several in vitro, in vivo, ex vivo, and human studies. Different types of extract mediums, forms, and phytochemicals of the plant have been analyzed in studies that are presented in the Section 6.1, Section 6.2, Section 6.3, Section 6.4, Section 6.5, Section 6.6, Section 6.7, Section 6.8, Section 6.9, Section 6.10, Section 6.11 and Section 6.12 (Figure 2).

### 6.1. Antioxidant and Antiaging Properties

Antioxidants are an important property which can be responsible for a range of positive outcomes in different diseases and conditions, such as cancer, asthma, diabetes, anti-aging treatments, immune modulation, and cosmetics [42,43,44,45,46,47]. The antioxidant properties of *P. erosus* compounds isolated from the peel and tuber extracts were reported through various solvents in different studies. The antioxidant properties of an ethyl acetate extract from the tuber of *P. erosus* (PTE) were assayed by measuring the scavenging activity against 1,1-diphenyl-1-picrylhydrazine (DPPH), which is a method to assess the antioxidant potential of samples. The four compounds (daidzein, daidzein-7-*O*-β-glucopyranose, 5-hydroxy-daidzein-7-*O*-β-glucopyranose, and 8,9-furanyl-pterocarpan-3-ol) isolated from the PTE extract were also studied for their antioxidant properties against DPPH and were found to be active [6,16]. In another study, a tyrosinase inhibition assay was also carried out on the extract, as well as other isolated compounds, which is the method to indicate the skin whitening property of compounds. The extract, as well as the compounds, had the potential activities needed to justify their use in cosmetics for skin whitening and anti-aging properties [6]. Later, researchers also carried out the antioxidant activities of water and alcohol PTE through DPPH and ABTS (2,2′-azinobis-(3-ethylbenzthiazolin-6-sulfonic Acid)) assay methods, which confirmed the extract’s potential as a cosmetic material that possesses anti-oxidant and anti-melanogenic activities [5]. Recently, a 70% ethanolic *P. erosus* tuber peel extract (PPE) and PTE were tested for antioxidant properties in a DPPH assay, as well as for skin whitening activities in a tyrosinase inhibition assay, which again confirmed the anti-aging and skin whitening properties of *P. erosus*. [7].

A limited study has been carried out to decipher the antioxidant mechanisms of the extracts/compounds from *P. erosus* in organisms. Recently, the treatment of a synbiotic drink made from *P. erosus* juice and kefir grains on Wistar rats (*Rattus norvegicus*) was studied for oxidative stress markers in the testicular tissue damage caused by hyperlipidemia. The study revealed that the oxidative stress marker malondialdehyde (MDA) was decreased and superoxide dismutase (SOD) was increased in the treatment group. An increase in SOD activity could be a possible mechanism of the antioxidant effects, but this study did not focus precisely on *P. erosus* and other factors, such as probiotics, were present in the study.

However, earlier studies on the antioxidant compounds found in *P. erosus* can provide insight into the potential antioxidant mechanisms that may be effective in the in vivo condition. The polyphenolic compounds (such as daidzein) can increase the expression of the proteins, mRNAs, and activities of SOD, which is an important antioxidant target which converts superoxide radicals into ordinary oxygen and hydrogen peroxide [48]. Similarly, vitexin is found to exert its antioxidant effects through the reduction in the level of reactive oxygen species (ROS) and MDA inside the cells, which is attributed to the increase in the activity of antioxidant enzymes like SOD, glutathione, heme oxygenase, and NAD(P)H quinone oxidoreductase 1. It also upregulates the antioxidant response proteins, such as AMP-activated protein kinases and nuclear factor erythroid 2-related factor 2 [49,50,51,52]. Metal chelation through the polyphenolic compound of *P. erosus* can also be an important mechanism for antioxidant activity. Polyphenols can chelate metal at physiological pH values. Hence, most polyphenols are effective metal chelators in the biological system [53]. The polyphenols reported from *P. erosus* can decrease the production of hydroxyl radicals in the Fenton reaction through the chelation of metal ions such as Fe^2+^, which is the required substrate in the reaction [54].

Importantly, the antioxidant activity of *P. erosus* may be one of the important reasons for most of its biological properties, such as its anti-diabetes, anticancer, immune modulating, and gastric ulcer preventions reported from the extracts/compounds of *P. erosus* [42,43,44,45,46].

### 6.2. Anti-Diabetes

In 2017, it was estimated that more than 450 million adults had diabetes, and the figure is expected to increase without effective control measures [55]. Different approaches have been used to manage this global disease [56,57,58]. Phytochemicals with pharmacological properties have gained momentum against diabetes in the recent past [59]. The anti-diabetes activities of the tuber and seeds in different extracts and forms from *P. erosus* have been studied in different experiments. Researchers have identified the α-glucosidase and α-amylase inhibitory activities of the tuber extract (PTE) in in vitro assays (Table 2). In vivo studies on male ICR mice showed the normalization effect of the postprandial glucose response in normal, as well as diabetic, mice (Table 3) [60]. In a different study on male C57BL/KsJ-db/db mice, the administration of aqueous PTE found potential anti-diabetes effects on different parameters such as hemoglobin A1c (HbA1c), and plasma insulin values were found to be significantly reduced. The hepatic glycogen concentration was found to be increased and the intraperitoneal glucose tolerance test was also found to be improved [61]. A study on male Bagg and Albino (BALB/c) mice with a treatment of tuber fibers resulted in a significant reduction in blood glucose levels [62]. In a recent study, the pancreatic tissue was also found to be protected from high fat diet-induced islet hyperplasia and hypertrophy in BALB mice treated with tuber powder [63,64]. Probiotic yogurt made from the *P. erosus* tuber was also found to be effective in decreasing blood glucose in DDY strain mice when compared with normal probiotic yogurt [65].

The important factors for anti-diabetes properties can be attributed to the presence of soluble fibers which include inulin [66], as well as other phytochemicals that are present in the tuber. Dietary inulin has also shown anti-diabetes effects in different studies [67,68].

However, directly drinking the tuber juice of *P. erosus*, which is rich in carbohydrates (such as fructose and glucose) can cause an increase in blood sugar just after the administration [69].

### 6.3. Anticancer

Cancer is still one of the leading causes of death globally and new cases of cancer patients increased from approximately 18 million in 2018 to potentially more than 23 million new cases per year in 2030 [70]. Synthetic drugs can have major disadvantages, which include drug resistance, side effects, toxicity to normal tissues, and the recurrence of cancer. Phytochemicals are a promising option, as they may have fewer side effects, and there is evidence that phytochemicals can target a wide variety of pathways/signaling pathways in growth, proliferation, differentiation, cell cycles, and apoptosis [71,72,73,74,75,76].

The anticancer properties of seeds from *P. erosus* were primarily studied due to the presence of rotenone and similar phytochemicals which were known to have anticancer effects [77]. Recently, the protein present in the seeds was also found to be a highly potent anticancer agent [11].

The anticancer activities of compounds isolated from the seeds were studied in different cancer cell lines. Rotenone and hydroxy-rotenone isolated from the seeds were found to exhibit potent cytotoxic effects on a number of tumor cell lines, such as P-388 in lymphocytic leukemia, KB in carcinoma of the nasopharynx, a multidrug-resistant variant of KB, KB-V1, and a number of human cancer cell lines derived from a variety of tumor types, namely, fibrosarcoma, lung cancer, colon cancer, melanomas, and breast carcinoma [10,78]. Significant cytotoxic activity was observed from rotenone isolated from *P. erosus* seeds on K562 human leukemia cells in MTT assays [38]. Although the toxicity of rotenone and other similar compounds on normal cells can be the major bottleneck in the development of anticancer drugs, recently the radiolytic derivative of rotenone (rotenoisin A) was developed which was safe to use on primary epidermal keratinocytes (no inhibitory effects, as with rotenone) and showed the inhibition of breast cancer cell proliferation, as well as enhanced apoptosis [79]. Similarly, the radiolytic derivative of rotenone (Rotenoisin B) was found to have low toxic effects on normal cells but strong anticancer effects on hepatic cancer through the inhibition of hepatic cancer cell proliferation and an increase in the apoptosis (Table 2) [80].

Researchers have also identified a novel ribosome inactivating protein (pachyerosin) from the seeds of *P. erosus*, which was found to be cytotoxic against the human hepatoma cell line, HuH-7. Furthermore, the immunotoxin prepared from pachyerosin has shown extremely high cytotoxicity in nano-molar concentrations [11]. These results from the different compounds and proteins of the seeds make them attractive candidates against cancer, which can be further pursued in anticancer drug development.

### 6.4. Immune Modulation

Immune modulation is one of the most important properties of *P. erosus*, which can be utilized to develop it as important intervention against different diseases. Immune modulation can have a positive effect on a number of disease conditions, including cancer and infectious diseases [81,82,83]. The immune modulatory effects of *P. erosus* was observed in in vitro, in vivo and ex vivo experiments (Table 2 and Table 3).

The *P. erosus* tuber fiber extract (PTFE) stimulated the production of TNF-α and interlukene-6 (IL-6) through the enhanced gene expression of both the proteins in cells (J774.1 cells) [84]. In the study, an anti-inflammatory cytokine, IL-10, was also found to be increased, which can prevent the damaging effects of macrophages from too much activation [85]. Immune-mediated genes, such as iNOS and COX-2, which are expressed in activated macrophages, were also found to be increased in PTFE treated cells in a dose-dependent manner, as compared to untreated cells. Similarly, increased levels of TNF-α and interlukene-6 (IL-6) were found in ex vivo and in vivo experiments with peritoneal macrophage (P-Mac) cells and BALB/c mice, respectively [84]. In a recent study, the fiber extract fractions (FEF) were studied for immunomodulatory effects on mouse peritoneal macrophages, lymphocytes, and cytokines. The immune-enhancing effect was observed with FEF treatment with increased phagocytic activity and a stimulation of both TNF-α and IL-6 production [12].

In another study, the IgM production by HB4C5 cells was found to be increased with the application of PTFE in a dose-dependent manner. Furthermore, the in vitro production of IgM, IgG, and IgA was studied in primary splenocytes from the BALB/c mice and was found to increase in a dose-dependent manner (Table 2). In the in vivo study, the oral application of PTFE resulted in increased serum immunoglobin levels and an enhanced production of immunoglobulin and cytokines from the lymphocytes of the spleen, Peyer’s patches, and the mesenteric lymph nodes in BALB/c mice [86].

In a recent study, the oral administration of methanolic extracts and ethyl ether fraction from the tuber of *P. erosus* was found to enhance the innate immunity and IgG production in mice already immunized with the Hepatitis B vaccine (HBV) [17]. In a similar study, the in vivo immune modulation activity of water soluble PTF from *P. erosus* was observed in BALB/c mice. An increase in phagocytotic macrophages, TNF-alpha production, NO production from the peritoneal macrophages, and lymphocyte proliferation was found with the administration of PTFE after the HBV [15] (Table 2 and Table 3).

### 6.5. Anti Herpes Simplex Virus (HSV)

Plants and their preparations have been used in folk medicine for antiviral treatments [87]. Some of these natural preparations have been found to target virus replication for antivirus activity [88,89]. Phytochemicals are supposed to have antiviral properties against different viral diseases, with superior pharmacokinetics and low side effects, and are considered a potential intervention against current and emerging viral diseases, including SARS-CoV-2 [90,91,92]. To evaluate antiviral properties, the researchers isolated nine compounds from the different chemical classes, such as iso-flavonoids, monosaccharides, and rotenoids from the seeds of *P. erosus* (Table 2). Two compounds (12a-hydroxydo-lineone and 12a-hydroxypachyrrhizone) belonging to the rotenoids class were found to have anti-herpes simplex virus activity in a plaque reduction assay [9].

### 6.6. Antifungal Activity

Fungi are eukaryotic organisms, so the development of antifungal drugs is more complicated as compared to antibacterial drugs. Therefore, fewer antifungal drugs have been discovered and are available against fungi compared to bacteria. Further drug resistance against antifungal drugs worsens the current scenario for the treatment of fungal diseases and phytochemicals are still the main resource of new antifungal drugs [93]. The antifungal activities were identified from the powder and extracts of the seeds from *P. erosus* in different studies. The isolated secondary metabolites from the extracts with antifungal properties were then characterized. The antifungal properties were carried out through mycelial inhibition bioassays on three important fungi, named *Colletotrichum gloeosporioides*, *Fusarium oxysporum*, and *Rhizopus stolonifera* (Table 2). The maximum fungicidal effects were attained with rotenone on *R. stolonifer*, with pachyrrizine on *F. oxysporum*, and with dehydroneotenone on *C. gloeosporioides* [13]. Further studies are required to develop these compounds (rotenone, dehydroneotenone, and pachyrrizine) as potential drug candidates against fungal diseases. Antifungal proteins were also reported from the seeds of *P. erosus*, which included PaAFP and the dimeric plant defensin protein SPE10 [94,95,96].

### 6.7. Phytoestrogenic Potential

Phytoestrogens are potential candidates for the prevention of cardiovascular diseases and postmenopausal osteoporosis [97,98]. Tuber juice from *P. erosus* was found to have estrogenic potential in different studies on animals [14,41,99,100]. The estrogenic activity may be due to the compounds present in the tuber juice, such as genistein and daidzein, that structurally resemble estrogen. The rats treated with *P. erosus* juice for 24 days had significant myometrium proliferation compared to the non-treated group [41]. Similar experiments in mice treated with *P. erosus* tubers for 24 days resulted in the proliferation of both secondary and tertiary uterine follicles [99]. In a recent study, rats treated with depo-medroxyprogesterone acetate (DMPA), which causes a hypoestrogenic response, were orally administered an ethanol extract of tubers (ETE). The significant increase in the number of antral follicles and endometrial stromal follicles were observed in the ETE treated groups (Table 3). The treated rats also showed an increased amount of endometrial epithelium in the study [100]. In these studies, it was suggested that the natural estrogenic role of *P. erosus* can be further developed to fulfill the need for estrogen therapies for premenopausal and postmenopausal women.

### 6.8. Anti-Osteoporosis Potential

Ovariectomized rat models of osteoporosis were used to explore the anti-osteoporosis effects of the ethyl acetate extract of the *P. erosus* tuber (PTE). An increase in bone density and mineral content, such as calcium and phosphorus in bone ash, was observed in the PTE administered group (Table 3). An increase in the bone length of the femur and tibiae were also reported in the treatment group and the ultimate load and stiffness of the femur was also significantly increased in the mechanical tests [14]. Researchers proposed that the phytoestrogenic compounds present in the PTE might be responsible for the activity and they suggested the potential of PTE medicinal extracts against osteoporosis in post-menopausal women.

### 6.9. The Promotion of Cardiovascular Health

Cardiovascular diseases are the most important concern for human health and are responsible for the highest mortality in the world [101]. The cardiovascular health-promoting effects of *P. erosus* tuber juice (PTJ) was studied in healthy volunteers for its effects on their heart rate, systolic and diastolic blood pressure, serum K^+^ concentrations, ex vivo platelet aggregation, and plasma cGMP concentrations. PTJ attenuated ex vivo collagen-induced platelet aggregation and also reduced the diastolic blood pressure in the healthy volunteers (Table 3) [102]. Dietary nitrates present in PTJ can be absorbed after ingestion, converted to nitrite, and further converted to NO, which may attenuate platelet responses to collagen stimulation.

In the recent preliminary research on hyperlipidemic rats treated with the synbiotic drink from PTJ concentrate, the MDA level in the heart tissue of the rats improved. The study suggests the potential role of PTJ in lowering the risk of hypertension [103]. Further research is required to understand the molecular mechanisms and to develop PTJ as a therapeutic candidate or prophylactic intervention for cardiovascular health.

### 6.10. Central Nervous System Depressant Activity

Central nervous system depressants (CNSDs) are an important class of drugs. Phytochemicals that, as natural compounds, may possess superior properties for blood–brain barrier penetration which makes them better candidates for CNSDs [104]. The researchers explored the CNSD activity of seeds from *P. erosus*, as in folk medicine these seeds were used to treat insomnia. The ethanolic extract of seeds was used in mice to test locomotor activity, muscle coordination, behaviors, sleep, and anxiety (Table 3). The results from study revealed that the seed extract produced muscle relaxation effects, reduced locomotor activity, and increased antianxiety and anti-aggressive activities [8]. In different experiments, CNSD activity was observed; however, more detailed studies are required to explore the potential of *P. erosus* for CNSD activity.

### 6.11. Preventive Effects in Gastric Ulcers

Considering the antioxidant effects of *P. erosus*, the juice of the tuber has been evaluated as a preventive agent on alcohol-induced gastric ulcers in mice [105]. A high consumption of alcohol is known to impair the gastric mucosal barrier, causing extensive hemorrhagic injuries, an accumulation of oxidative stress, and increased inflammation [106]. Different doses of juice from *P. erosus* and *Raphanus sativus* separately and in combination were given to mice in the experiments. Tuber juice from *P. erosus* was found to be the most effective in the prevention of gastric ulcers in histopathology and in index values of gastric ulcers [105].

### 6.12. Insecticidal Activity

The insecticidal activity of extracts from the seeds and leaves of *P. erosus* was studied against different insects [107,108]. The long-term oviposition deterrent activity of the seed extract was observed in the diamondback moth (*Plutella xylostella*) on its plant host, which was maintained for a long time due to the low volatility of the extract [108]. In another study, water and ethanolic extracts of seeds of *P. erosus* had the highest (compared to two other plants, i.e., *Hyptis suaveolens* and *Apium graveolens*) insecticidal activity against the larvae and adults of *Aedes aegypti*, which is the dengue virus vector [109]. Similarly, insecticidal activity against odorous house ants (*Tapinoma sessile*) was found to be better than arrowhead (*Syngonium podophyllum*) (Table 3). Researchers found the insecticidal activity of extracts from *P. erosus* highly promising, and also proposed the need to further extend the investigation of insecticidal activity against other important insects [107,108].

## 7. Gaps and Future Directions

*P. erosus* has shown positive outcomes for a number of diseases, along with its use as a food crop, which makes this plant highly important, demanding further research and development efforts to establish the medicinal/health supplement properties for possible health-promoting usages. A high nutritional profile with nitrogen-fixing properties and the biodiesel potential of the seed oil are important reasons to increase its production in different countries. This also highlights the requirement to expedite the directional research efforts to develop the pharmacological applications of the plant. However, the different medicinal properties of the plant have different possible challenges.

The antioxidant and immune modulation properties of the *P. erosus* were found in several studies, which can strongly establish these findings. These properties can be taken as the key prospective biological properties of the plant, as these biological properties have positive effects against several diseases. Immune modulation has been studied in in vivo, in vitro, and ex vivo experiments, but like other known biological properties (such as anticancer, antiviral, and antifungal properties) the compounds responsible for immune modulation are not well known/studied. Additionally, the molecular mechanisms for immune modulation of the *P. erosus* extracts are also not well studied, which can hinder its development as a therapeutic for immune modulation. Furthermore, a major limitation is the absence of human/clinical studies to establish the immune modulation properties of *P. erosus*. Clinical trials/studies are, therefore, required and proposed for its development as a therapeutic and/or as a food supplement for immune modulation.

In the case of the antioxidant properties, which have been already utilized for cosmetics in different products [111], as for immune modulation, the compounds for antioxidant activity are not well studied/known. Studies on the responsible compounds and molecular mechanisms of antioxidants must be pursued in the near future for the optimal utilization of antioxidant properties, not only in cosmetics, but also for anti-aging and to treat other important diseases.

The anti-diabetes properties of *P. erosus* was also apparent in several studies (Table 2 and Table 3). The soluble fibers, such as inulin, are one of the main suspected constituents that can be considered as the reason for its anti-diabetes properties [67,68]. However, anti-diabetes compounds/constituents are not precisely studied from *P. erosus* and are yet to be discovered. The important gap which must be filled for the development of *P. erosus* as an anti-diabetes intervention are clinical/human studies, which must be carried out as a high priority. Different studies on animals have supported its anti-diabetes potential and the long-term usage of the tuber as food in different countries have abolished the potential risk in clinical/human studies. However, the sudden increase in blood glucose levels with the intake of juice from the tuber is reported; therefore, tuber juice, if used in diabetic subjects, might require special precautions.

The major challenge to develop anticancer drugs from *P. erosus* is the toxicity of known anticancer compounds used from the seeds of *P. erosus*, which can be overcome with the discovery of nontoxic constituents important for anticancer activities from *P. erosus*. Secondly, the researchers have been preparing less toxic/nontoxic derivatives of the toxic constituent rotenone. Anticancer studies of *P. erosus* have been mostly limited to various cell lines, which must be first addressed in in vivo and mechanistic experiments before clinical studies.

The antimicrobial potential against antifungal and antiviral activities were discovered and the compounds of the activities were also identified (Table 2). Antifungal proteins were also identified from the plant [94,95,96]. The development of *P. erosus* (powder, extract, or the extracted compound) as an anti-microbial therapy requires further research efforts, which include the study of the antimicrobial potential against other infectious diseases and the safe study of seed extracts and compounds. Additional in vivo studies would again be required before clinical anti-microbial studies.

The phytoestrogen properties of the tuber of *P. erosus* were established through different in vivo studies on the myometrium. Furthermore, phytoestrogenic effects such as anti-osteoporosis effects on ovariectomized rats and the potential cardio protective effects on volunteers were also observed. Compounds with similar structures to estrogen present in tuber juice are suspected to be the reason for the phytoestrogenic properties, but further studies are required to confirm the phytoconstituent responsible for the effect and molecular mechanism of action. Clinical studies can also be used to explore the phytoestrogenic potential of the tuber.

Initial indications of the cardio protective potential of the *P. erosus* tuber in human and ex vivo experiments have paved the path to the development of *P. erosus* as a cardio protective supplement/medication. These initial findings demand more experiments to be carried out to explore its cardio protective properties. Dietary nitrate is suspected to be the reason for the cardio protective properties that again requires further experiments to validate these speculations. The discovery of phytoconstituents responsible would be important to establish the tuber as an intervention for cardio protection through further studies. Similarly, CNSD activity was apparent from different experiments, such as a study on mice models, but more studies must be repeated on different animals before considering this for further clinical studies. The toxicity of the seeds again could be the major bottleneck in in vivo studies before clinical studies, as the toxicity of seeds is reported in different cases. The discovery of the phytochemical that is responsible for CNSD activities could be a step towards harvesting the potential of *P. erosus* as a CNSD therapeutic.

Broadly, it can be suggested that clinical studies are required for the further development of most of the biological properties of the *P. erosus* tuber, such as its anti-diabetes, anti-osteoporosis, cardio protective, immune modulation, antioxidant, and anti-aging properties. In the case of the biological properties from the seeds, such as the anticancer, antiviral, antifungal, and CNSD properties, safety studies before human/clinical studies would be required as the toxic effects of the seeds are already reported [23,24,25,26].

It is also suggested that *P. erosus* may also have effective anti-obesity properties, which can be discovered in the near future, as the anti-diabetes effects may also help to control weight [112,113]. In a study on the anti-diabetes effects of *P. erosus*, the anti-obesity effect was also observed in the mice [62]. In recent research, a study has been conducted to analyze the potential of *P. erosus* tuber fiber in the dysregulation of energy metabolism and adiposity in adult male BALB/c mice fed with a high-fat diet. The results support the potential of *P. erosus* tuber fiber as a supplement to minimize the disruption of energy homeostasis and obesity [114]. Research focused on the anti-obesity effects of extracts and/or phytoconstituents from *P. erosus* are also suggested to be carried out in different experiments.

Several compounds, such as dulcitol, gentisic acid, formononetin, kaikasaponin III, p-coumaric acid, and vitexin were known to be present in *P. erosus* but were not tested/studied in the context of the biological activity of *P. erosus* (Table 1). These compounds have been known to possess important biological activities [115,116,117,118,119,120] that are suggested to be explored accordingly in *P. erosus*.

Recently, dulcitol, which was reported to be present in the seeds of *P. erosus*, was found to have anticancer activity on cell lines and in in vivo experiments [119,121]. Similarly, formononetin, the compound present in the leaves of *P. erosus*, is also known for its anticancer potential through several possible molecular targets [118,122]. Therefore, the anticancer potential of the leaves from *P. erosus* is suggested to be explored in anticancer experiments.

Recently, wound healing through gentisic acid (a compound present in the leaves) was reported [120], although other biological properties of gentisic acid, such as its neuro-protective, anti-microbial, anti-oxidant, anti-inflammatory, antigenotoxic, and hepatoprotective activities were also reported in the literature [123]. Similarly, vitexin, which is present in the leaves of *P. erosus*, also possesses different biological properties, such as neuro-protective, anticancer, anti-inflammatory, and anti-hyperalgesic properties [115]. As per the presence of both of these active compounds, the associated biological properties, especially the neuro-protective properties, can be explored in future studies of the leaves of *P. erosus*.

It can be concluded that, like its utilization as a food crop, the huge potential of the phytochemicals and the biological properties present in *P. erosus* are still underutilized. The suggested directions of action from the current review can boost and utilize the potential of *P. erosus*, not only in promoting health, but also in the pharmaceutical and cosmetics industries.

## Figures and Tables

**Table 1:** Phytochemicals with potential antioxidant properties identified in *P. erosus*.

Sr.	Name	Type	Amount/Yield	Sample	Activity	References
1	12a-Hydroxydolineone	Rotenoids	31 μg/g	Seeds	Against HSV types 1 and 2	[9,10]
2	12a-Hydroxyerosone	Rotenoids	6 μg/g	Seeds	NA	[10]
3	12a-hydroxypachyrhizone	Rotenoids	36 μg/g	Seeds	Against HSV types 1 and 2	[9,10,38]
4	12a-Hydroxyrotenone	Rotenoids	9 μg/g	Seeds	Anticancer and against HSV types 1 and 2	[9,10]
5	Dehydroneotenone	Isoflavone	4 μg/g	Seeds	Antifungal activity	[9,10,13]
6	Dolineone	Rotenoids	60 μg/g	Seeds	Antifungal activity	[9,13,38]
7	Erosone	Isoflavonoids		Seeds	Antifungal activity	[13]
8	Munduserone	Rotenoids		Seeds	NA	[10]
9	Neodulin	3-arylcoumarin (pterocarpan)	3.4% μg/g	Seeds	NA	[10]
10	Neotenone	Isoflavonoids	41 μg/g	Seeds	NA	[9]
11	Pachyrrhizine	Isoflavonoids	35 μg/g	Seeds Seeds Seeds	Antifungal activity	[9,10,13,38]
12	Pachyrrhizone	Rotenoids	117 μg/g	Seeds	Antifungal activity	[9,13]
13	Pachyrrhisomene	Pterocarpan	04.7 μg/g	Seeds	NA	[10]
14	Rotenone	Rotenoids	153 μg/g	Seeds	Anticancer and antifungal activity	[10,13,38]
15	(8,9)-Furanyl-pterocarpan-3-ol	Pterocarpan	141.05 μg/g	Tuber	Antioxidant and skin whitening	[6,37]
16	2-Butoxy-2,5-bis(hydroxymethyl)-tetrahydrofurane-3,4-diol	Other		Tuber	Antioxidant and skin whitening	[37]
17	4-(2-(Furane-2-yl)ethyl)-2-methyl-2,5- dihydro-furane-3-carbaldehyde	Other		Tuber	Antioxidant and skin whitening	[37]
18	5-Hydroxy-daidzein-7-O-*β*-glucopyranose	Isoflavonoids		Tuber	Skin whitening	[6]
19	Daidzein-7-*O*-β- glucopyranose /Daidzin	Isoflavonoids	329.93 ± 10.00 μg/g	Tuber	Antioxidant and skin whitening -	[4,6,35,37]
20	Genistin	Isoflavonoids		Tuber	Antioxidant and skin whitening	[37]
21	Kaikasaponin III	Triterpenoid glycosides (Saponin)	69 μg/g	Tuber	NA	[35]
22	Daidzein	Isoflavonoids	2356.10 ± 38.47 μg/g	Tuber juice	Antioxidant and skin whitening	[4,6,37,41]
23	Genistein	Isoflavonoids	1655.30 μg/g	Tuber juice	NA	[41]
24	Formononetin	7-hydroisoflavones	65.13 ± 3.01 μg/g	Leaves	NA	[4]
25	Gentisic acid	Dihydroxybenzoic acid	69.03 ± 1.00 μg/g	Leaves	NA	[4]
26	l-Phenylalanine	Amino acid	16,654.43 ± 43.52 μg/g	Leaves	NA	[4]
27	Malonyl genistin	Glycosyloxyisoflavone	683.77 ± 12.69 μg/g	Leaves	NA	[4]
28	Malonyldaidzin	Glycosyloxyisoflavone	9853.90 ± 44.28 μg/g	Leaves	NA	[4]
29	*p*-Coumaric acid	Aromatic acid	612.83 ± 2.02 μg/g	Leaves	NA	[4]
30	*p*-Hydroxybenzoic acid	Aromatic acid	161.63 ± 1.52 μg/g	Leaves	NA	[4]
31	Salicylic acid	Aromatic acid	58.83 ± 2.57 μg/g	Leaves	NA	[4]
32	Vitexin	Flavones	1970.12 ± 9.22 μg/g	Leaves	NA	[4]
33	Isobavachalcone	Flavonoids	34.3 μg/g	Leaves	Anticancer	[39]
34	Wighteone	Flavonoids	2.9 μg/g	Leaves	Anticancer	[39]
35	Prunin	Flavonoids	2.7 μg/g	Leaves	NA	[39]
36	Orientin	Flavonoids	50 μg/g	Leaves	NA	[39]
37	Erosusone	Flavonoids	2 μg/g	Leaves	Anticancer	[39]
38	3-episedumoside F_1_	Megastigmane glycoside epimer	20 μg/g	Leaves	NA	[39]

**Table 2:** Different biological activity through in vitro studies.

Sr. No.	Activity	Model/Method	Dose/Duration	Component Used	Result	Reference
1	Antioxidant/anti-aging	DPPH antioxidant assay	6.25–200 (µg/mL)	*P. erosus* peel ethanolic extract	IC_50_ = 84.09 ± 4.87 (µg/mL)	[7]
		DPPH antioxidant assay	6.25–200 (µm/mL)	*P. erosus* tuber ethanolic extract	IC_50_ = 98.30 ± 1.30 (µm/mL)	[7]
		Tyrosinase activity inhibition	3.13–100 (µg/mL)	*P. erosus* peel ethonolic extract	IC_50_ = 194.51 ± 7.63 (µm/mL)	[7]
		Tyrosinase activity inhibition	3.13–100 (µg/mL)	*P. erosus* tuber ethonolic extract	IC_50_ = 97.05 ± 0.86 (gm/mL)	[7]
		Scavenging activity on ABTS radical (IC_50_)	100 µL at different concentrations	*P. erosus* water extract	IC_50_ = 1825.16 ± 22.87 (µg/mL)	[5]
		Scavenging activity on ABTS radical (IC_50_)	100 µL at different concentrations	*P. erosus* (70%) ethanol extract	IC_50_ = 1711.71 ± 58.09 (µg/mL)	[5]
		DPPH antioxidant assay	100 µL at different concentrations	*P. erosus* water extract	IC_50_ = 1215.65 ± 65.99 (µgm/mL)	[5]
		DPPH antioxidant assay	100 µL at different concentrations	*P. erosus* (70%) ethanol extract	IC_50_ = 998.10 ± 117.71(µg/mL)	[5]
2	Anti-diabetes	α-glucosidase inhibitory assay	0.05–0.25 (µg/mL)	*P. erosus* tuber extract	IC_50_ = 0.083 ± 0.004 (mg/mL)	[60]
		α-amylase inhibitory activity	0.05–0.25 (µg/mL)	*P. erosus* tuber extract	IC_50_ = 0.091 ± 0.017 (mg/mL)	[60]
3	Immune modulation	ELISA is used to measure antibodies on HB4C5 cells and splenocytes	Sample conc. 0.1–100 (mg/mL)	*P. erosus* tuber fiber extract	The production levels of immunoglobulin, i.e., IgM, IgG, and IgA, as well as cytokines, were significantly enhanced	[86]
		Phagocytosis activity J774.1 cell/P-Mac cells	1.69, 6.75, and 27 mg/mL	*P. erosus* tuber fiber extract	An increase in phagocytosis activity and the production of pro-inflammatory cytokines was observed	[84]
4	Antiviral	Plaque reduction assay was performed for HSV-1 (KOS) or HSV-2	100 µL at different concentrations	Compounds isolated from seeds	12a-hydroxydolineone and 12a-hydroxypachyrrhizone found to have activity against both HSV-1 and -2	[9]
5	Antifungal	Growth inhibitory effects on fungi	0.5, 2.0, 5.0, and 10 mg/mL	*P. erosus* seed powder	−2 to −15% (inhibition)	[13]
		Growth inhibitory effects on fungi	2.0, 5.0 and 10 mg/mL	*P. erosus* seed extract with hexane, dicloromethane, and acetone	5.4 to −64.9% (inhibition)	[13]
		Growth inhibitory effects on fungi	250 µg/mL	Compounds isolated from seeds	2.81–56.2% (inhibition)	[13]
		Inhibitory activities on the growth of different fungi were determined by microspectrophotometry	15 μg/ml	SPE10 (a dimeric plant defensin protein) from the seeds of *P. erosus*	IC_50_ of 15 μg/mL (most effective against *Bipolaris maydis*)	[95]
		Inhibitory activities on the growth of fungi (*Trichoderma viride* and *Chrysosporium luteum*)	6.2–12.5 μg	PaAFP (the protein isolated and purified from the *P. erosus* seeds	*Trichoderma viride* and *Chrysosporium luteum* at 6.2 μg and 12.5 μg per disc, respectively, inhibited growth	[94]
6	Anticancer	Human hepatoma cell line HuH-7	0.06 mL/well	Pachyerosin protein isolated from seeds of *P. erosus*	IC_50_ of 0.050 ± 0.004 nM for pachyerosin(immunotoxin) and IC_50_ of 117.92 ± 10.21 nM for pachyerosin	[11]
		The viability of K562 cells determined by the MTT assay	0.06 mL/well	*P. erosus* seed extract (acetone) and compound rotenone from seed extract	The *P. erosus* extract and rotenone displayed IC_50_ 40.5 mg/mL and 13.05 mM, respectively	[38]

**Table 3:** Different biological activities of *P. erosus* through in vivo, ex vivo, and human studies.

Sr. No.	Activity	Model	Dose Duration	Substance	Result	References
1	Anti-diabetes	Male ICR mice induced for diabetes mellitus by streptozotocin Blood glucose was measured using a glucometer	(200 mg/kg) at 0, 30, 60, and 120 min	*P. erosus* tuber extract	The AUC for the glucose response of *P. erosus* extract-fed group (658.1 ± 18.0 mg·h/dL) was lower than that of the control group (742.5 ± 24.7 mg·h/dL) (*p* < 0.05)	[60]
		Male C57BL/KsJ-db/db mice; blood glucose and glycosylated hemoglobin levels	(0.5 g/100 g diet) 6 weeks	*P. erosus* tuber extract in water	HbA1c values for *P. erosus* extract-fed group (9.11 ± 1.06) was lower than that of the control group (12.92 ± 0.31) in db/db control groups (*p* < 0.05)	[61]
		Plasma insulin level	(0.5 g/100 g diet) 6 weeks	*P. erosus* tuber extract in water	Plasma insulin values for *P. erosus* extract-fed group (191.85 ± 4.65) was lower than that of the control group (251.04 ± 25.24) in db/db-control groups (*p* < 0.05)	[61]
		Homeostatic index of insulin resistance (HOMA-IR) and quantitative insulin sensitivity check index (QUICKI)	(0.5 g/100 g diet) 6 weeks	*P. erosus* tuber extract in water	HOMA-IR values for *P. erosus* extract-fed group (22.26 ± 0.53) was lower than that of the control group (40.43 ± 4.03) in db/db-control groups (*p* < 0.05). QUICKI values for *P. erosus* extract fed group (0.24 ± 0.01) was higher than that of the control group (0.22 ± 0.01) in db/db-control groups (*p* < 0.05)	[61]
		Intraperitoneal glucose tolerance test (IPGTT)	(0.5 g/100 g diet) 6 weeks	*P. erosus* tuber extract in water	IPGTT outcomes were improved in db/db-JCE group mice compared to db/db-control group mice	[61]
		Hepatic glycogen assay	(0.5 g/100 g diet) 6 weeks	*P. erosus* tuber extract in water	Hepatic glycogen concentration values for *P. erosus* extract-fed group (116.49 ± 5.12) were higher than that of the control group (90.25 ± 4.75 mg/g) in db/db-control groups (*p* < 0.05)	[61]
		Male Bagg and Albino (BALB)/c mice fed with HSD Blood glucose measurements	10% and 25% *P. erosus* tuber Fiber (PTF) in diet 8over 8 weeks	*P. erosus* tuber fiber	Blood glucose level was significantly lower in JF groups (HSD + JF 10%, and HSD + JF 25%) as compared with HSD group, starting at 4th week of treatment (*p* < 0.05)	[62]
		Body weight and adipose tissue measurements	10% and 25% PTF in 8-week diet	*P. erosus* tuber fiber	The body weight gain was significantly lower in JF 25% group, but not in JF 10% group, as compared with HSD group (*p* < 0.01)	[62]
		24 male DDY strain mice	Yogurt, 2 mL for 7 days	Probiotic treatment group vs. using *P. erosus*, yogurt and control	*P. erosus* probiotic yogurt more effectively decreased (337.57 ± 90.01 mg / dL) blood glucose levels than the probiotic yogurt (*p* = 0.00)	[65]
		BALB (Bagg and Albino)/c mice	10% and 25% with high-fat diet for 8 weeks	*P. erosus* tuber fiber powder	Blood glucose was significantly lower in the treated groups and normal group. The pancreatic tissue was protected from high-fat diet induced islet hyperplasia and hypertrophy in mice treated the treatment group	[63,64]
		40–50-year-old people; 10 people as a case group and 10 people as a control group	Dose of 250 g (150 mL) per day given for 7 days	*P. erosus* starch extract	259.90 mg/dL, then 185.40 mg	[110]
		Male and female Wistar strain rats; hampering blood glucose in rat models	7 mL juice with glucose (50% concentration 2.5 gm/kg)	*P. erosus* tuber juice	The mean increase of blood glucose level was 324.45 mg/dL	[69]
2	Immune modulation	Male BALB/c mice induced with hepatitis B vaccine	25, 50, and 100 mg/kg *P. erosus* tuber fiber extract for 18 days	*P. erosus* tuber fiber extract	Treatment stimulated phagocytotic macrophages, NO production from peritoneal macrophages, and in vivo lymphocyte proliferation. Enhancements in phagocytic capacity index characterized by increased NO, IL-10, and TNF-production	[15]
		Male BALB/c mice induced with hepatitis B vaccine	100 and 200 mg/kg for 18 days	Methanolic and ethyl ether fraction (FAEF) from the tuber of *P. erosus*	Enhanced the innate immune response and IgG production. The immune enhancing effect was observed with FAEF treatment with increased phagocytic activity, and stimulation of both TNF-α and IL-6 production	[17]
		Female BALB/c mice	6.75 or 27 mg/kg body weight for 14 days	*P. erosus* tuber fiber extract	BFE may activate the adaptive immune responses by enhancing the production of Igs and cytokines in vivo	[86]
		BALB/c mice	6.75 mg/kg and 27 mg/kg for 7 days	*P. erosus* tuber fiber extract	BFE could activate macrophages by increasing the phagocytosis activity and production of pro-inflammatory cytokines in mouse P-Mac in vitro and in vivo	[84]
		BALB/c mice	6.75 and 27 mg/mL	Tuber fiber extract	Phagocytosis activity percentage of P-Mac ex vivo increased from 32.1 ± 1.9 to 42.6 ± 4.6 (*p* < 0.001)	[84]
3	Phytoesterogenic activity	Female Sprague–Dawley rat histopathology	1.5 mL *P. erosus* tuber juice for 24 days	*P. erosus* tuber juice	The myometrium in rats with *P. erosus* tuber juice treatment was wider than controls and pure daidzein treatment	[41]
		BALB/c female	0.3, 0.6, and 0.9 g/kg for 24 days	*P. erosus* tuber	Treatment caused proliferation of the uterine endometrium and myometrium layers and increased the number of uterine glands	[99]
		Rats (Female)	Three oral doses: 70, 140, and 280 mg/200 g every day for 14 days	Tuber ethanol extract	Significantly increased the number of antral follicles and endometrial stromal follicles; increased the amount of endometrial epithelium was observed	[100]
4	Anti-osteoporosis	Female Sprague–Dawley rats	200, 400, and 800 mg/kg for 28 days	Ethyl acetate extract of *P. erosus* tuber	Femoral length, tibiae length, bone density of bone ash; content of calcium, and bone ash content of phosphorus increased to 32.51 ± 0.92 mm, 37.25 ± 0.57 mm, 1.441 ± 0.064 g/cm^3^, 50.02 ± 6.55%, and 32.73 ± 1.09%, respectively	[14]
5	Cardio protection	Human platelet aggregation study and blood pressure measurement	500 mL	*P. erosus* tuber juice	The ex vivo collagen-induced platelet aggregation was significantly attenuated, as well as platelet inhibition and blood pressure lowering	[102]
6	Central nervous system depressant activity	Swiss albino mice of either sex	75 and 150 mg/kg	Ethanolic seed extract of *P. erosus*	Ethanolic extract of seeds of *P. erosus* possess sedative, anti-anxiety, muscle relaxant, and anti-aggressive properties in different experiments	[8]
7	Preventive effects on gastric ulcers	Swiss Webster mice	100, 300, and 600 mg/kg	*P. erosus* tuber juice	Reduced the number of ulcers, increased the ratio of protection, and repaired the cells in gastric histopathology	[105]
8	Insecticidal activity	Insecticide oviposition deterrent activity	0.1–2.0 (% *W*/*v*)	*P. erosus* seed extract in chloroform	Oviposition deterrent indices *P. erosus* seed extracts and coumarin partially deterred the diamondback moth adults from laying eggs on treated leaves in a concentration-dependent manner	[108]
		Larvae and adults of *Aedes aegypti*	Up to 100 mg/mL	*P. erosus* seed water and (70%) alcoholic extract	Lethal concentration 50 (LC_50_) of 16.22 ± 0.20 µg/mL and LC5_0_ value of 91.41 ± 0.49 µg/mL	[109]

## Supplementary Materials

The following supporting information can be downloaded at: https://www.mdpi.com/article/10.3390/antiox11010058/s1, Table S1: Volatile and other phytochemicals identified in *P. erosus*.
