# Supplementary material for: The Bioactivity and Phytochemicals of Pachyrhizus erosus (L.) Urb.: A Multifunctional Underutilized Crop Plant
Source: Antioxidants (Basel). 2021 Dec 27;11(1):58. doi: 10.3390/antiox11010058 (PMC8773301; doi:10.3390/antiox11010058)
Supplement: Supplementary file 1 [file antioxidants-11-00058-s001.zip › antioxidants-1498170-supplementary.pdf]

Supplementary Material

Table S1. Volatile and other phytochemicals identified in *P. erosus*.

Sr. No.	Name	Type	Amount/yield	Sample	Activity	Reference
1	Hexanoic acid 2- methylpropyl ester	Volatile compound (VC)	1.66% [1]	Leaves	NA	[40]
2	Pentanal	VC	1.66% [1]	Leaves	NA	[40]
3	Pentanol	VC	6.43% [1]	Leaves	NA	[40]
4	Furan 2-ethyl	VC	6.44% [1]	Leaves	NA	[40]
5	3-Hexen-1-ol (Z)	VC	10.57% [1]	Leaves	NA	[40]
6	Cyclohexanone	VC	32.84% [1]	Leaves	NA	[40]
7	3-hexenal (Z)	VC	32.71% [1]	Leaves	NA	[40]
8	(*E*,*E*)-2,4-Hexadienal	VC	0.07% [1]	Leaves	NA	[40]
9	Heptanal	VC	0.23% [1]	Leaves	NA	[40]
10	2-Heptenal	VC	3.84% [1]	Leaves	NA	[40]
11	Heptadienal, (*E*,*E*)-2-4	VC	1.96% [1]	Leaves	NA	[40]
12	(*E*)-3-octen-2-one	VC	0.51% [1]	Leaves	NA	[40]
13	(*E*)-2-octenal	VC	0.04% [1]	Leaves	NA	[40]
14	1-Octanol	VC	0.06% [1]	Leaves	NA	[40]
15	3-methyl-1- butanol	VC	0.18% [1]	Leaves	NA	[40]
16	2-Cyclohexen-1-one, 3- methyl-6-(1-methylethyl)	VC	0.08% [1]	Leaves	NA	[40]
17	2-Methylpropan oic acid 3-hydroxy-2,4,4- trimethylpenty l ester	VC	0.02% [1]	Leaves	NA	[40]
18	(*E*)-*β*-Ionone sesquiterpene	VC	0.06% [1]	Leaves	NA	[40]
19	3,5,9-Undecatrien-2- one,6,10- dimethylterpene	VC	0.10% [1]	Leaves	NA	[40]
20	2-Methylpropan oic acid,1-(1,1-dimethylethyl)-2-methyl-1,3- propanediyl ester	VC	0.08% [1]	Leaves	NA	[40]
21	Sedumoside F_1	Megastigmanes	12 μg/g	Leaves	NA	[39]
22	(3S,5R,6S,9R)-3,6-dihydroxy-5,6-dihydro-β-ionol	Megastigmanes	30.4 μg/g	Leaves	NA	[39]
23	4,5-dihydroblumenol A	Megastigmanes	60.6 μg/g	Leaves	NA	[39]
24	Ampelopsisionoside	Megastigmanes	70 μg/g	Leaves	NA	[39]
25	Simplicifloranoside	Megastigmanes	32.4 μg/g	Leaves	NA	[39]
26	(6S,9R)-roseoside	Megastigmanes	45.2 μg/g	Leaves	NA	[39]
27	3-(2′,3′-dihydroxy-4′-hydroxymethyltetrahydrofuran-1′-yl) pyridine-4,5-diol	pyridine-4,5-diol derivative	7.2 μg/g	Leaves	NA	[39]
28	1,4-methano-3-benzo[d]oxepin-2(1H)-one	3-benzoxepine lactone	9 μg/g	Leaves	NA	[39]
29	3-episedumoside F_1	Megastigmane glycoside epimer	20 μg/g	Leaves	NA	[39]
30	Phaseoside IV	triterpenoid glycosides	23 μg/g	Tuber	NA	[35]
31	Dulcitol	Monosaccharide	95 μg/g	Seeds	NA	[9]

[1] Relative percentage of the total peak area.
